# MicroRNA-184 promotes apoptosis of trophoblast cells via targeting WIG1 and induces early spontaneous abortion

**DOI:** 10.1038/s41419-019-1443-2

**Published:** 2019-03-04

**Authors:** Yuan Zhang, Ji Zhou, Ming-qing Li, Jie Xu, Jin-ping Zhang, Li-ping Jin

**Affiliations:** 1 Clinical and Translational Research Center, Shanghai First Maternity and Infant Hospital, Tongji University School of Medicine, Shanghai, 201204 People’s Republic of China; 2 Institutes of Biology and Medical Sciences, Soochow University, Suzhou, Jiangsu Province 215123 People’s Republic of China; 3 Laboratory for Reproductive Immunology, Hospital of Obstetrics and Gynecology, Fudan University Shanghai Medical College, Shanghai, 200011 People’s Republic of China

## Abstract

Recurrent spontaneous abortion (RSA) refers to the unintentional termination of two or more consecutive pregnancies that severely threatens human reproductive health. Our previous study has shown that miR-184 is expressed more highly in RSA than in normal pregnancy, whether in the villus or decidua. In this study, compared with normal pregnant women, the expression of miR-184 in decidual stromal cells (DSCs) and decidual immune cells (DICs), as well as in peripheral blood, from RSA patients was enhanced similarly. Moreover, we found miR-184 could promote the apoptosis and repress the proliferation of trophoblast cells. Further exploration indicated that miR-184 upregulated the expression of Fas by targeting WIG1 thus inducing cell apoptosis. Finally, after miR-184 overexpression in vivo, the embryo resorption rate in pregnant mice was increased significantly. Therefore, our study outlines the pivotal role of miR-184 in maintaining successful pregnancy, providing a new diagnostic and therapeutic target for RSA.

## Introduction

As an embryo allograft, successful pregnancy needs the maternal immune system to recognize but not reject the fetal alloantigen^1^. Threatening 1–5% of women of reproductive age, recurrent spontaneous abortion is defined as two or more consecutive spontaneous abortions, which has increasingly affected human reproductive health^2^. Other than known pathogenic factors, including chromosomal abnormalities, endocrinological factors, and immune dysfunction, still almost half of the causes of RSA are unclear and further explanation is urgently needed^3^.

As the main constituent cells of human placenta, embryo-derived trophoblast cells proliferate, differentiate, and invade the uterine endometrium via a series of processes, regulated exquisitely through intercellular signaling mediated by hormones, cytokines, and growth factors^4^. Certainly, trophoblast cells elicit a variety of biological functions at the maternal-fetal interface, involving anchorage of the placenta, reshaping of maternal spiral arteries, modulation of decidual angiogenesis, secretion of hormones and cytokines, and crosstalk with maternal immune cells. Deficiency in the function of trophoblast cells could result in serious complications of human pregnancy, such as pregnancy loss, preeclampsia, and intrauterine growth restriction^1,5^. As another important component of placenta, decidua is composed of decidual stromal cells (DSCs) and decidual immune cells (DICs). These immune cells, including decidual natural killer (NK) cells, macrophages, T cells and dendritic cells (DCs), must work together to maintain immune tolerance at the maternal-fetal interface^6,7^.

MicroRNAs (miRNAs) are a group of small non-coding RNAs composed of 20–24 nucleotides. By binding to the 3’ untranslated region (3’ UTR) of target messenger RNAs (mRNAs), miRNAs induce target mRNA degradation or inhibit its translation, thus participate in a wide range of biologic and pathologic processes, such as cell differentiation, proliferation, apoptosis, angiogenesis, and even inflammation^8,9^. Previous studies have found that abnormal expression of miRNAs is closely related to reproductive system diseases, including endometriosis, preeclampsia, and infertility. For example, CYR61, a key regulator for wound recovery, tumor growth, vascular disease, and embryo development, could be repressed by miR-155 and then lead to preeclampsia^10^.

Recently, several studies have shown that miRNAs are critical for the maintenance of normal pregnancy by regulating the differentiation, proliferation, invasion, and even apoptosis of trophoblast cells, thus becoming a research hotspot in recurrent spontaneous abortion. MiR-16 can inhibit placental angiogenesis by reducing the expression of vascular endothelial growth factor (VEGF), resulting in spontaneous miscarriage^11^. In addition, it has been demonstrated that circulating miRNAs in the plasma may serve as early predictive noninvasive biomarkers of unexplained recurrent spontaneous abortion (URSA)^12^. Moreover, our previous study has indicated that miR-184 is highly expressed in decidua and villus from recurrent spontaneous abortion patients^13^, suggesting that miR-184 may be involved in the development of a successful pregnancy. Therefore, the current study was undertaken to investigate the related mechanisms to reveal the role of miR-184 in pregnancy.

## Materials and methods

### Specimen collection

All tissue samples were collected with informed consent according to the requirements of the Research Ethics Committee in Shanghai First Maternity and Infant Hospital, Tongji University School of Medicine. All subjects completed informed consent forms for collection of tissue samples. Similarly, the current study was specifically approved by the Research Ethics Committee.

Normal decidua samples were obtained from normal pregnant women (age 29.24 ± 3.17 years; gestational age 8.11 ± 1.37 weeks), who terminated pregnancy for non-medical reasons.

Decidua samples of RSA were obtained from patients (age 28.37 ± 1.46 years; gestational age 7.53 ± 1.52 weeks), who had two or more URSAs, as well as excluded other causes, such as reproductive malformation, infection, and chromosome abnormality.

The peripheral blood of RSA (age 28.78 ± 2.39 years; gestational age 8.63 ± 1.21 weeks) was also collected according to the aforementioned standards, and the peripheral blood of the control group was collected from normal pregnant women (age 29.24 ± 3.17 years; gestational age 8.11 ± 1.37 weeks).

Villi tissues from normal pregnant women (age 30.62 ± 1.147 years; gestational age 7.615 ± 0.3676 weeks) and RSA patients (age 32.31 ± 1.046 years; gestational age 7.538 ± 0.3859 weeks) were achieved complying with the above standards.

### Isolation and culture of primary cells

The decidual tissues from the first-trimester pregnancy were quickly placed into cold DMEM/F12, transported to the laboratory within 1 h after surgery, and washed with Hank balanced salt solution for the isolation of DSCs and DICs, the process of which was handled according to our previous procedures^14^.

### Total RNA extraction and qRT-PCR

Total RNAs of cells or tissues were purified by TRIzol reagent (Takara), followed by reversely transcripted into cDNA with a reverse transcription kit (Takara) according to the manufacturer’s description. qRT-PCR was carried out using FastStart Universal SYBR Green Master (Roche Diagnostics) and analyzed using the Real-Time Detection System (Eppendorf, Hauppauge, NY, USA). The polymerase chain reaction (PCR) parameters were 95 °C 10 min, 95 °C 15 s, 60 °C 30 s, and the melting curve. The relative primers as followed: miR-184 RT: 5′-CTCAACTGGTGTCGTGGAGTCGGCAATTCAGTTGAGACCCTTAT-3′; miR-184-F: 5′-ACACTCCAGCTGGGTGGACGGAGAACTGAT-3′; miR-184-R: 5′-CTCAACTGGTGTCGTGGA-3′; β-actin-F: 5′-GGCACCACCATGTACCCTGGCAT-3′; β-actin-R: 5′-TCCTGCTTGCTGATCCACATCTGCT-3′.

### Plasmid construction

A fragment of the hsa-miR-184 gene was amplified from human genomic DNA by PCR with the following primers: F: 5′-CCGCTCGAGCAAGTGGAGATCTCACTGAC-3′, R: 5′-CCGGAATTCCGGTAGCTGTCCAGAGCTGC-3′. The fragment was cloned into a vector of pMDH1-PGK-GFP using the sites of *Xho*I and *Eco*RI, and the insert sequence was further confirmed by sequencing.

### Luciferase assay

The predicted wild-type or mutant 3’-UTRs of the target gene containing has-miR-184 binding sites were constructed by ABM Inc (Zhenjiang, Jiangsu, China). To evaluate the binding activity between hsa-miR-184 and 3’-UTR of target genes, approximate 10^5^ 293T cells per well in a 24-well plate were transiently transfected with 0.5 μg of each firefly luciferase reporter construct, 0.1 μg of Renilla luciferase TK vector, and 1.0 μg of pMDH1-PGK-GFP-miR-184. pMDH1-PGK-GFP vector was used as a control. TK vector was used to normalize transfection efficiency. Forty-eight hours after transfection, both firefly and Renilla luciferase activity were assayed. Normalized relative units represent firefly luciferase activity/Renilla luciferase activity.

### Immunoblot analysis

The relative cells or tissues were lysed with RIPA lysis buffer containing 1 mM PMSF for 15 min on ice. 3 X SDS loading buffer containing 15% β-mercaptoethanol was added into the supernatants of lysis. The mixture was boiled for 10 min and subjected to 10–15% SDS–PAGE gel with the same amount of total proteins. Then the gel was transferred to nitrocellulose membrane by electro-blotting. Proteins were detected with 1:1000 dilution of Fas, FasL (Abcam, Cambridge, MA, USA), caspase-3, -7, -8, and -9, AGO2, LASP1, TMED2, FOXO3a, FOXP3, PDX1, MAFG (Cell Signaling Technology, Danvers, MA, USA), WIG-1 (Proteintech, Chicago, IL, USA), and 1:5000 dilution of HRP-linked anti-rabbit antibody (Cell Signaling Technology, Danvers, MA, USA) and HRP-conjugated donkey anti-mouse antibody (Proteintech, Chicago, IL, USA). GAPDH was used as loading reference.

### Cell culture and transfection

HTR8 cell line was cultured in DMEM/F12 (HyClone, Logan, UT, USA) with 10% FBS (Gibco, Hong Kong) and 1% penicillin–streptomycin solution. 293T cell line was cultured in DMEM (HyClone) with 10% FBS (Gibco) and 1% penicillin–streptomycin solution. The relative small interfering RNAs (siRNAs) (purchased from Ribobio Biotech) were transfected into HTR8 cells by using Lipofectamine 2000 (Invitrogen, Waltham, MA, USA) according to the manufacturer’s instruction. Protein levels were detected after 48 h.

To generate hsa-miR-184 overexpression cells, HTR8 cell line was transfected by lentivirus encoding hsa-miR-184 and its control lentivirus purchased from Suzhou GenePharma. After 48 h, the GFP-positive cells were sorted by FACS AriaIII (BD, Franklin Lakes, NJ, USA).

### Apoptosis assay

For apoptosis assay, HTR8 cells were digested by 0.25% trypsin without EDTA. Then, single-cell suspensions were stained with APC-Annexin V and propidium iodide in Annexin V binding buffer (BioLegend) according to the protocol. Cells were analyzed using BD FACS CantoII. FACS data were analyzed using FlowJo software (Tree Star).

To inducing the evident apoptosis of HTR8 cells, cells were planted in 24-well plates coated by 1–5 μg/mL purified anti-CD95 antibody (BioLegend) and cultured for 24 h. Then these cells were collected and detected.

### Proliferation assay

For Cell Counting Kit (CCK-8) assay, relative cells were planted in 96-well plates and cultured for 48 h. After adding 10 μL CCK-8 (Sangon Biotech, Shanghai, China) into every well and culturing for 2 h, absorbance value was detected by using Synergy2 microplate reader (BioTek, Winooski, VT, USA).

### Cell invasion assay

As described in our previous study, 15 μL of pure Matrigel (BD Bioscience) was added to the upper surface of the filter in transwell plates, with air-drying under sterile conditions. Then, Matrigel was rehydrated with 100 μL of warm DMEM/F12 for 2 h, followed by seeding the HTR8 cells in the upper chamber (2 × 10^5^ resuspended in 200 μL of DMEM/F12 without FBS), while 600 μL of DMEM/F12 supplemented with 10% FBS was added into the plates. After culturing for 48 h, cells attaching to the upper surface of the filter were removed with a cotton swab, and cells remaining on the lower surface were fixed in methanol and stained with hematoxylin in turn. Thereafter, cells migrating to the lower surface were counted under a light microscope in five random fields at a magnification of × 200.

### Mice

All the mice used in our experiments were BALB/c (6 to 8 weeks of age) and kept in a specific pathogen-free animal facility. Female mice were injected with miR-184 agomir or negative control agomir (purchased from Ribobio Biotech) via tail vein every 3 days until sacrificed. After the first injection, female mice were mated with male mice. Eleven days after seeing a plug, the female mice were killed and the embryo resorption rate was calculated.

### TUNEL assay

For the apoptosis assay of tissue, placental sections of mice were treated with fluorescence-based TUNEL kit (Roche, Basel, Swiss) according to the manufacturer’s instructions. Photographs were obtained with a fluorescence microscope (Nikon, Tokyo, Japan).

### Statistical analysis

Unpaired student’s *t*-test (two-tailed) or two-way ANOVA was used for comparisons. All statistical analyses were performed using GraphPad Prism 6. Data represent mean ± SEM. *P* < 0.05 was considered statistically significant.

## Results

### The expression of miR-184 is increased in RSA patients

**Fig. 1:**
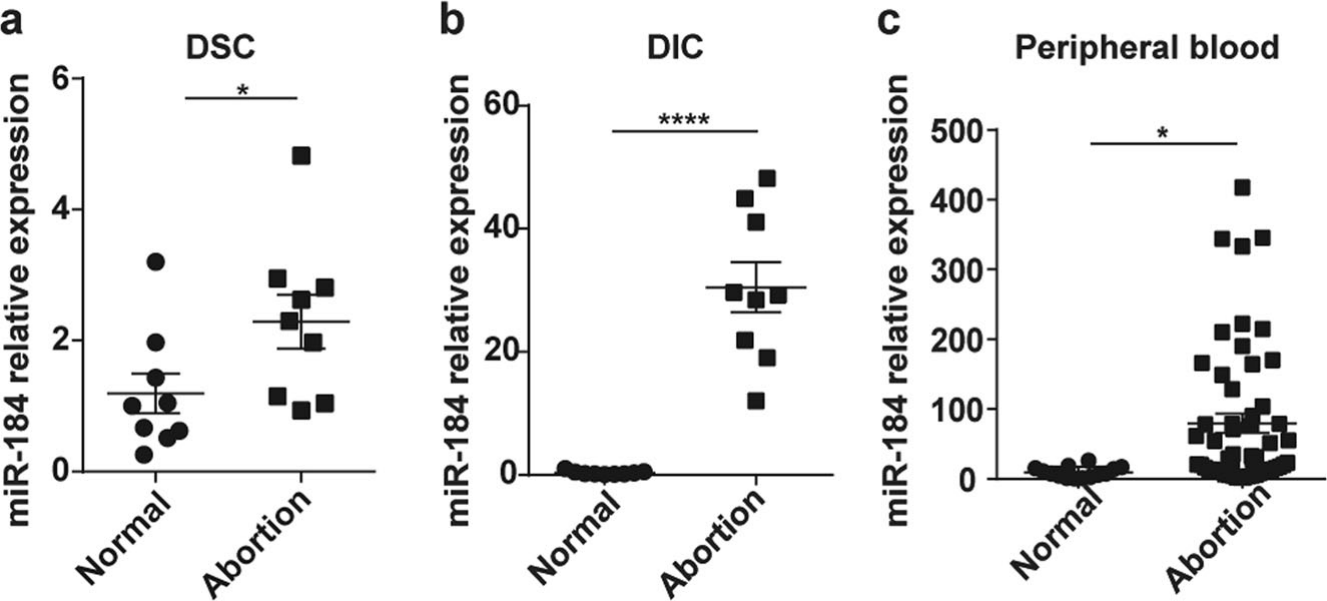
The expression of miR-184 is increased in RSA patients. **a** Comparison of miR-184 expression in DSCs between normal pregnant women and abortion patients. Unpaired student’s *t*-test (two-tailed) was used for comparison between two groups. *P < 0.05. Data represent mean ± standard error of the mean [SEM]. (*n* = 9) **b** Comparison of miR-184 expression in DICs between normal pregnant women and abortion patients. Unpaired student’s *t*-test (two-tailed) was used for comparison between two groups. ****P < 0.0001. Data represent mean ± SEM. (*n* = 9) **c** Comparison of miR-184 expression in the plasma of peripheral blood between normal pregnant women (*n* = 14) and abortion patients (*n* = 54). Unpaired student’s *t*-test (two-tailed) was used for comparison between two groups. *P < 0.05. Data represent mean ± SEM. (normal: normal pregnancy, abortion: RSA)

Based on the expression profiling of miRNAs at the maternal-fetal interface^11^, miR-184 exists in both the decidua and villus, the expression of which was higher in RSA than in normal pregnancy. After isolation from the decidua of RSA and normal pregnancy, the expression of miR-184 in DSCs and DICs was examined by real-time polymerase chain reaction (RT-PCR), confirming that miR-184 was highly expressed in DSCs and DICs of RSA (Fig. 1a, b). Additionally, expression of miR-184 in the peripheral blood of RSA and normal pregnancy was detected as well (GMINIX), the result of which was surprisingly consistent with that at the maternal-fetal interface (Fig. 1c), thus giving miR-184 a novel biomarker for the diagnosis of RSA.

### MiR-184 promotes the apoptosis of trophoblast cell

**Fig. 2:**
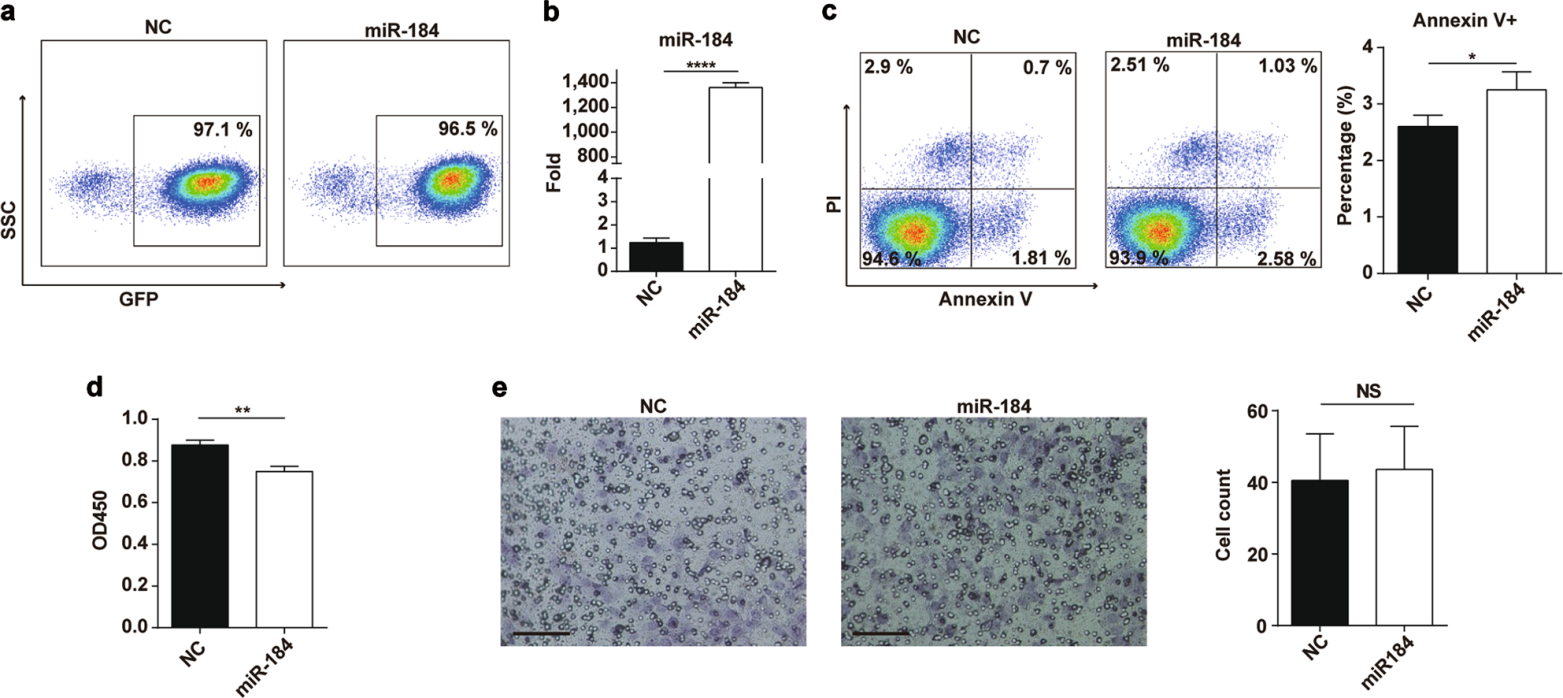
MiR-184 promotes the apoptosis and inhibits the proliferation of trophoblast cells. **a** HTR8 cells were infected with negative control lentivirus and miR-184 overexpression lentivirus for 72 h, and GFP-positive cells were sorted out. Flow cytometry was used to detect the infection efficiency. **b** We also used RT-PCR to detect the mRNA expression of miR-184 in NC and miR-184 cells. Unpaired student’s *t*-test (two-tailed) was used for comparison between two groups. ****P < 0.0001. Data represent mean ± SEM. **c** CCK8 was used to detect cell proliferation of NC and miR-184 cells, which indicated that miR-184 could markedly inhibit cell proliferation. Unpaired student’s *t*-test (two-tailed) was used for comparison between two groups. **P < 0.01. Data represent mean ± SEM. **d** After culturing NC and miR-184 cells for 48 h, the Annexin V staining result showed that miR-184 could increase cell apoptosis significantly. Unpaired student’s *t*-test (two-tailed) was used for comparison between two groups. *P < 0.05. Data represent mean ± SEM. **e** We examined the invasiveness of NC and miR-184 cells via transwell assay, finding that miR-184 had little effect on the invasiveness of trophoblast cells. Scale bar: 100 μm. Unpaired student’s *t*-test (two-tailed) was used for comparison between two groups. Data represent mean ± SEM. These experiments were repeated three times. (miR-184: HTR8 cells infected with miR-184 overexpression lentivirus; NC: HTR8 cells infected with negative control lentivirus)

To explore the effects of miR-184 on the biological functions of trophoblast cells, we infected HTR8 cells with negative control and miR-184 overexpression lentivirus, and sorted out GFP-positive cells to culture. The infection efficiency was measured by RT-PCR and flow cytometry (Fig. 2a, b), indicating that miR-184 was indeed overexpressed in HTR8 cells. Next, through detecting the proliferation, apoptosis, and invasiveness of infected HTR8 cells, we found that miR-184 could suppress cell proliferation (Fig. 2c) and promote the apoptosis of HTR8 cells significantly (Fig. 2d), but had no effect on cell invasiveness (Fig. 2e). Given these findings, we speculated that cell proliferation was inhibited as a result of the enhanced cell apoptosis.

### MiR-184 upregulates the expression of Fas in HTR8 cells

**Fig. 3:**
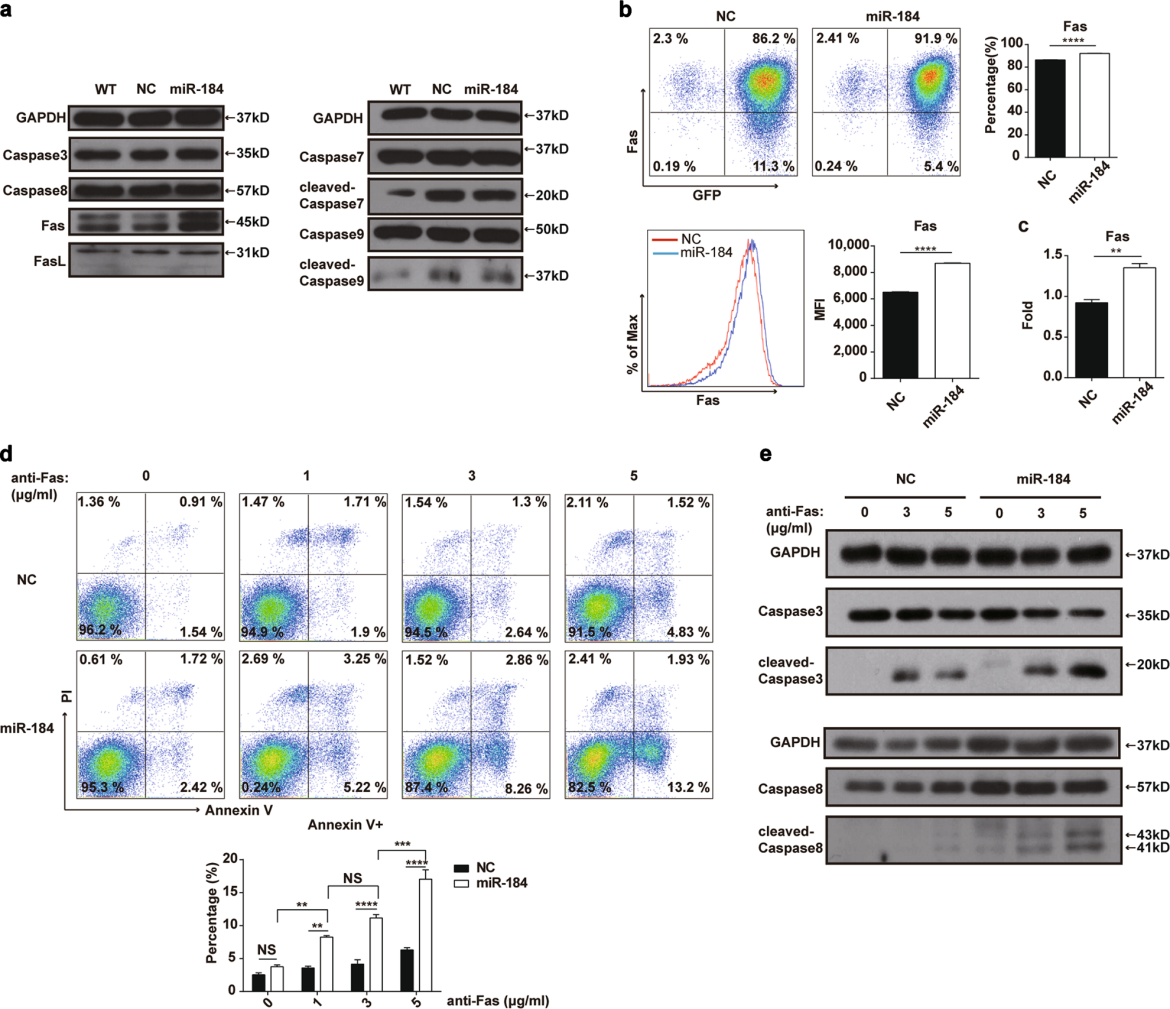
MiR-184 upregulates the expression of Fas in HTR8 cells. **a** We detected some molecules related to the poptotic pathway by western blot analysis, including caspase-9, -8, -3, -7, Fas, and FasL, and only the expression of Fas was changed after miR-184 overexpression. **b** Flow cytometry was used to examine the expression of Fas on NC and miR-184 cells, showing that the percentage of Fas-positive cells was increased in miR-184 cells, as well as the mean fluorescence intensity. Unpaired student’s *t*-test (two-tailed) was used for comparison between two groups. ****P < 0.0001. Data represent mean ± SEM. **c** Meanwhile, RT-PCR was applied to detect the mRNA level of Fas in two groups. Unpaired student’s *t*-test (two-tailed) was used for comparison between two groups. **P < 0.01. Data represent mean ± SEM. **d** After stimulation with various concentrations of anti-Fas activating antibody (zero, 1, 3, 5 μg /mL) for 24 h, the apoptosis rate of NC and miR-184 cells was detected. Two-way ANOVA was used for comparison between these groups. **P < 0.01, ***P < 0.001, ****P < 0.0001. Data represent mean ± SEM. **e** The protein level of caspase-3, -8, and cleaved caspase-3, -8 in NC and miR-184 cells after stimulation with different concentrations of anti-Fas activating antibody (zero, 3, 5 μg /mL) was also examined by western blot analysis. These experiments were repeated three times. (miR-184: HTR8 cells infected with miR-184 overexpression lentivirus; NC: HTR8 cells infected with negative control lentivirus.)

Then, further exploration was conducted to determine the related signal pathway in the aforementioned cell apoptosis. Western blot analysis was used to detect some molecules involved in the apoptotic signaling pathway, including Fas, caspase-3, caspase-8, and so on, the result of which showed that the protein level of Fas was increased in HTR8 cells after miR-184 overexpression, whereas the activation of related caspase molecules was not detected (Fig. 3a). Then, by flow cytometry, expression of Fas on HTR8 cells was confirmed. As shown in Fig. 3b, miR-184 enhanced the expression of Fas on HTR8 cells, no matter the percentage of Fas-positive cells or the mean fluorescence intensity. In addition, the mRNA level of Fas in HTR8 cells was also measured by RT-PCR, which was consistent with the protein level (Fig. 3c). Moreover, to further verify the aforementioned findings, anti-Fas activating antibody was used to induce the apoptosis of NC and miR-184 cells with the concentration varying from 0 to 5 μg/mL, and we found that the percentage of Annexin V^+^ miR-184 cells was higher than that of NC cells in a dose-dependent manner (Fig. 3d). In addition, caspase-8 and caspase-3 were activated after stimulation with different concentrations of anti-Fas activating antibody (Fig. 3e). Therefore, miR-184 could upregulate the expression of Fas in HTR8 cells, that in turn accelerates cell apoptosis.

### WIG1 is a functional target gene of miR-184

**Fig. 4:**
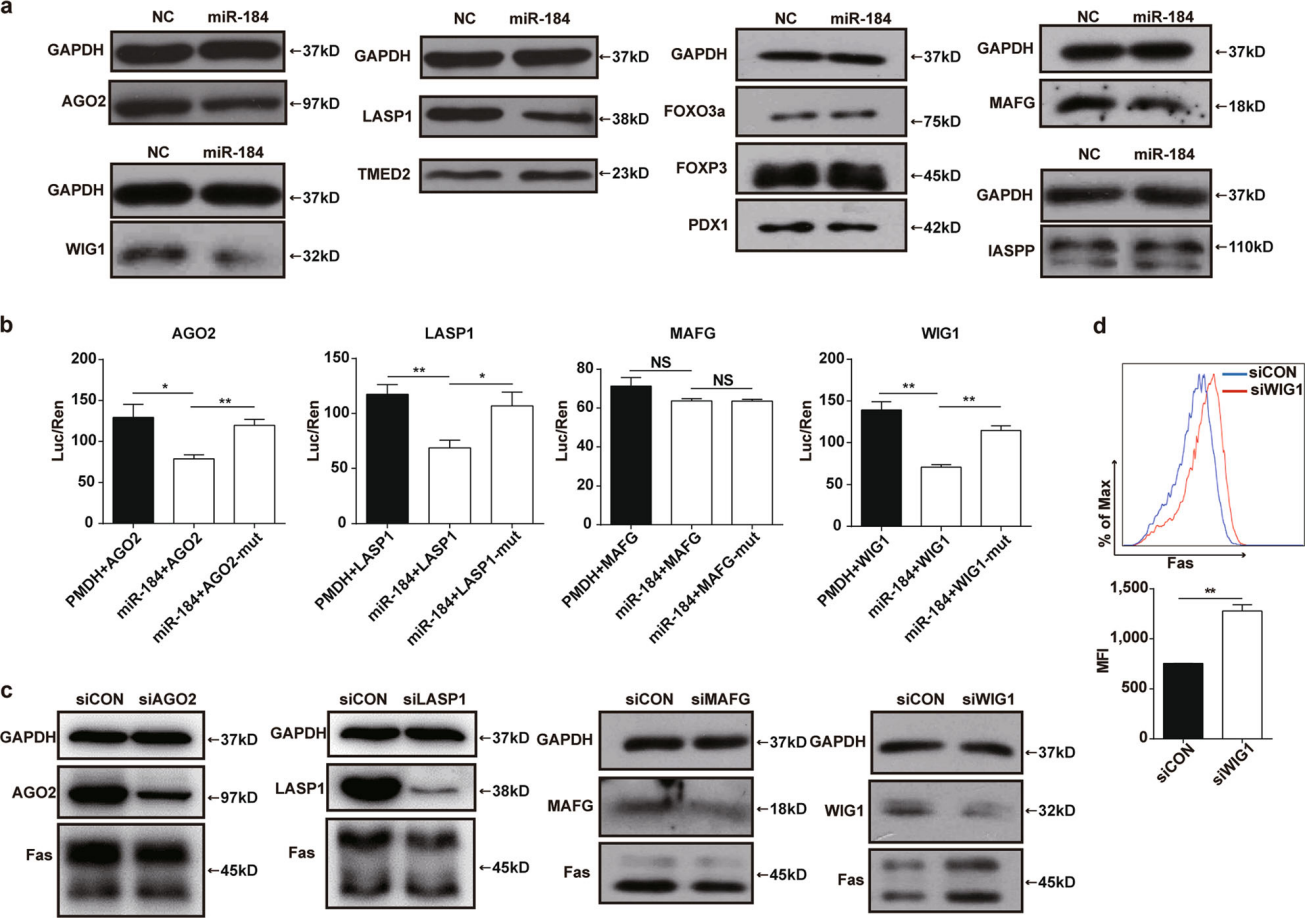
WIG1 (ZMAT3) is a functional target gene of miR-184. **a** We detected the protein levels of AGO2, LASP1, TMED2, FOXP3, Pdx1 FoxO3a, MAFG, iASPP, and WIG1 in NC and miR-184 overexpression cells via western blot analysis, and found that only the expression of AGO2, LASP1, MAFG, and WIG1 was decreased in miR-184 overexpression cells. **b** Luciferase reporter vectors containing the wild-type or mutant 3’UTRs of AGO2, LASP1, MAFG, and WIG1 were cotransfected with miR-184 plasmid into HEK293T cells for 48 h, and luciferase reporter assays were performed to define the target genes. Unpaired student’s t-test (two-tailed) was used for comparison between two groups. *P < 0.05, **P < 0.01. Data represent mean ± SEM. **c** After transfection with siRNAs for 48 h, expression of AGO2, LASP1, MAFG, WIG1 and the expression of Fas in HTR8 cells was detected via western blot analysis. **d** Flow cytometry was used to confirm the result obtained from western blot analysis. Unpaired student’s *t*-test (two-tailed) was used for comparison between two groups. **P < 0.01. Data represent mean ± SEM. These experiments were repeated three times

To identify the molecular mechanism by which miR-184 upregulates the expression of Fas in HTR8 cells, bioinformatic analysis was executed to predict potential targets of miR-184 using the miRWalk website (http://zmf.umm.uni-heidelberg.de/apps/zmf/mirwalk/). In addition, mass spectrometry and the JASPAR software (http://jaspar.genereg.net/) were used for target gene prediction. Ultimately, we selected several potential target genes to verify. The protein levels of AGO2, LASP1, TMED2, FOXP3, Pdx1 FoxO3a, MAFG, iASPP, and WIG1 in NC and miR-184 overexpression cells were analyzed by western blot, suggesting that the protein levels of AGO2, LASP1, MAFG, and WIG1 were decreased after miR-184 overexpression, whereas others had no significant difference (Fig. 4a). Meanwhile, luciferase reporter assays showed that luciferase activity was inhibited after transfecting the wild-type 3’UTRs of AGO2, LASP1, and WIG1 with miR-184 plasmid in HEK293T cells, whereas no significant changes were observed in those transfected with their corresponding mutant 3’UTRs. However, cotransfecting wild-type or mutant 3’UTR of MAFG with miR-184 plasmid had no obvious effect on the luciferase activity. (Fig. 4b) Next, to confirm the real target of miR-184 affecting the expression of Fas, siRNAs were applied to reduce the expression of the four genes in HTR8 cells. Results of western blot analysis elucidated that these genes were knocked down efficiently by their own siRNAs. However, the protein level of Fas was only increased significantly when WIG1 was decreased, whereas there was no change in other conditions (Fig. 4c), showing that only WIG1 was a functional target gene of miR-184. In addition, we also detected the expression of Fas on HTR8 cells infected with siRNA via flow cytometry, the result of which was consistent with the protein level as expected (Fig. 4d). Taken together, WIG1 is a functional target gene of miR-184 to upregulate the expression of Fas, thereafter inducing the apoptosis of trophoblast cells.

### MiR-184 increases the embryo resorption rate in vivo

**Fig. 5:**
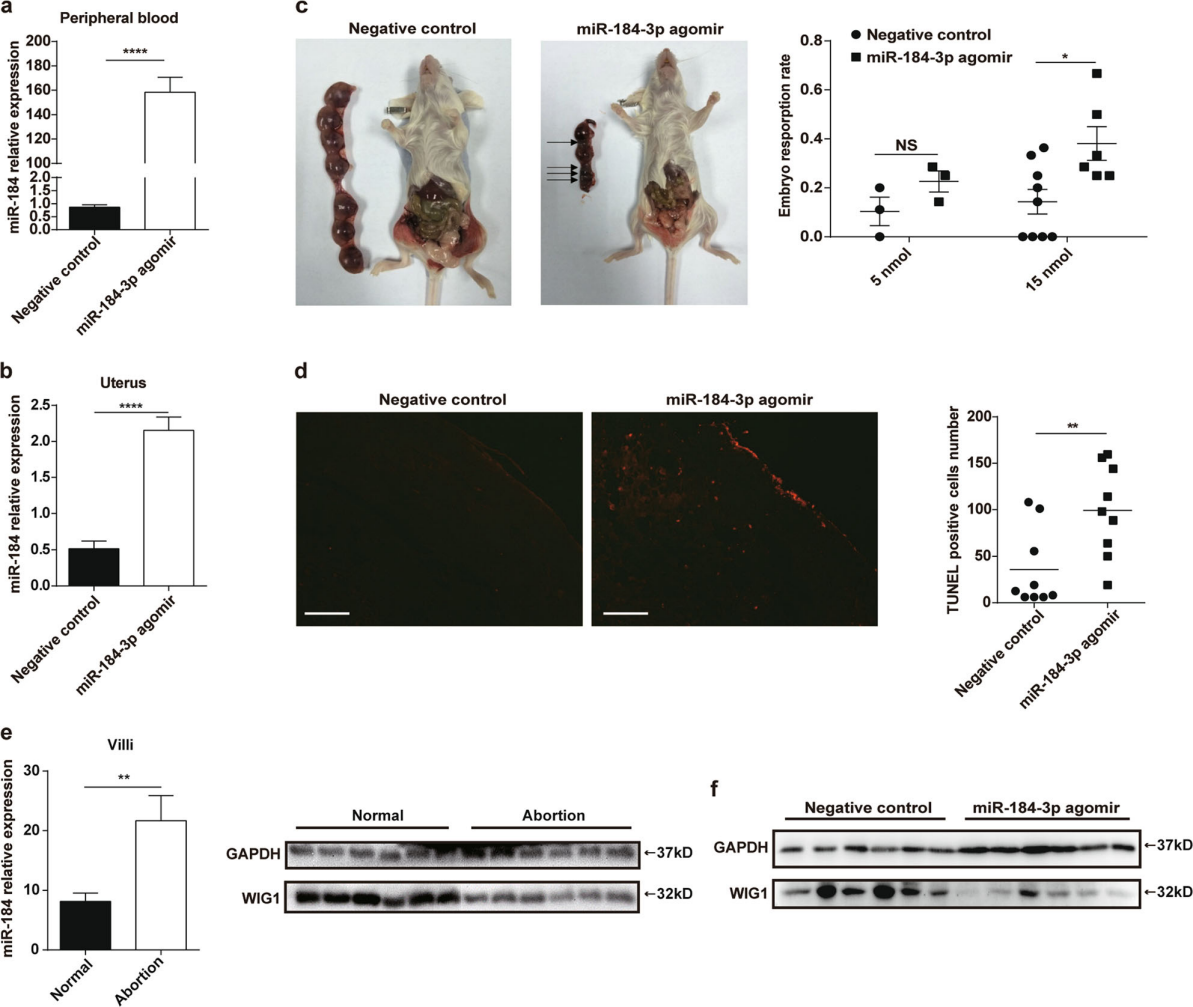
MiR-184 increases the embryo resorption rate in vivo. **a** In two groups of mice, we administered 15 nmol negative control and miR-184-3p agomir respectively, and detected the expression of miR-184 in the peripheral blood of these mice 24 h later. Unpaired student’s *t*-test (two-tailed) was used for comparison between two groups. ****P < 0.0001. Data represent mean ± SEM. **b** At the 11th day of pregnancy, we detected miR-184 expression in uteruses from pregnant mice. Unpaired student’s *t*-test (two-tailed) was used for comparison between two groups. ****P < 0.0001. Data represent mean ± SEM. **c** In two groups of mice, we administered negative control or miR-184-3p agomir once every 3 days respectively, and executed pregnant mice at the 11th day of pregnancy. Although administration with 5 nmol miR-184-3p agomir could increase the embryo resorption rate, there was no obvious difference between these two groups. However, administration with 15 nmol miR-184-3p agomir could significantly increase the embryo resorption rate. Arrows indicate the embryo resorption. Two-way ANOVA was used for comparison between the two groups. *P < 0.05. Data represent mean ± SEM. **d** TUNEL assay was used to compare the apoptosis of placental tissues between the two groups. Scale bar: 100 μm. Unpaired student’s t-test (two-tailed) was used for comparison between two groups. **P < 0.01. Data represent mean ± SEM. **e** Relative expression of miR-184 in villi tissues from normal pregnant women and abortion patients was detected by RT-PCR. (*n* = 13) And western blot was used to evaluate the expression of WIG1 in villi tissues from normal pregnant women and abortion patients. (*n* = 6) Unpaired student’s *t*-test (two-tailed) was used for comparison between two groups. **P < 0.01. Data represent mean ± SEM. **f** Expression of WIG1 was also tested in placental tissues from BALB/c mice receiving either negative control or miR-184-3p agomir. (*n* = 6) (Negative control: mice injected with negative control; miR-184-3p agomir: mice injected with miR-184-3p agomir.)

To verify the role of miR-184 in vivo, female BALB/c mice received negative control and miR-184-3p agomir respectively to construct miR-184 overexpression model, and the expression of miR-184 in the peripheral blood was detected by RT-PCR. The result suggested that miR-184 was indeed overexpressed in mice injected with miR-184-3p agomir (Fig. 5a). Expression of miR-184 in uteruses was also examined, the result of which demonstrated that miR-184 was overexpressed similarly in uteruses of mice injected with miR-184-3p agomir (Fig. 5b). Thereafter, as shown in Fig. 5c, administration of miR-184-3p agomir could significantly increase the embryo resorption rate. Meanwhile, TUNEL assay was used to detect the apoptosis of placental tissues, and the result showed that miR-184 could accelerate the apoptosis of placenta (Fig. 5d). Finally, the expression level of WIG1 in villi samples and placentas of mice were verified. In Fig. 5e, the RT-PCR result demonstrated that expression of miR-184 in villi tissues from abortion patients was significantly higher than that from normal pregnant women. And, western blot was applied to detect the expression level of WIG1, result of which indicated that the expression of WIG1 was decreased in villi tissues from abortion patients (Fig. 5e). Similarly, the expression level of WIG1 was tested in placental tissues from BALB/c mice receiving either negative control or miR-184-3p agomir. The western blot result showed that expression of WIG1 was markedly reduced in placental tissues from BALB/c mice receiving miR-184-3p agomir (Fig. 5f). In brief, our study confirmed that miR-184 could induce trophoblast cell apoptosis by targeting WIG1 and increase embryo resorption rate of mice.

## Discussion

MiR-184 is located at the region 25.1 on the q-arm of chromosome 15^15^, the expression of which is tissue- and developmental stage-specific^16,17^. In mammals, mature miR-184 is generally expressed mainly in the brain, corneal epithelium, and testes^16,17^. As an oncogene or tumor suppressor, miR-184 has been reported to be involved in almost all fields of cancer biology, including cell proliferation, migration, and invasion. Abnormal expression of miR-184 was first found to promote cell proliferation and repress cell apoptosis in tongue carcinoma^18^. Later, in nasopharyngeal carcinoma, miR-184 could block cell growth and survival by directly targeting BCL2 and C-MYC^19^. However, the role of miR-184 at the maternal-fetal interface in recurrent spontaneous abortions is not so clear. Based on our previous findings, we further detected the expression of miR-184 in cell components of decidua, showing a significant increase in DSCs and DICs from abortion patients. Moreover, the expression of miR-184 in peripheral blood was consistent with that at the maternal-fetal interface, suggesting that miR-184 was indeed crucial for successful pregnancy.

In this study, we found that miR-184 could upregulate the expression of Fas on HTR8 cells by targeting WIG1, thus promoting cell apoptosis. Apoptosis, or programmed cell death, is critical for cell differentiation, removal of damaged cells, and stability of the immune system, which mainly contains two classic pathways^20^. To determine the signaling pathway in the current study, we detected the common molecules involved in cell apoptosis, and found that only the expression of Fas was changed, although little activation was observed in the downstream caspase family, possibly because of the limited Fas ligand (FasL) provided in the culture condition. Fas, also known as CD95/APO-1, belongs to the transmembrane protein family, and is a major member of the tumor necrosis factor superfamily^21^. Previous reports have demonstrated that iASPP, a p53 inhibitor, promotes apoptosis of retinal ganglion cells in a p53/Fas pathway-dependent manner^22^. In addition, miR-184 has been found to inhibit the proliferation and invasion of the central nervous system lymphoma cells by targeting iASPP^23^. In addition, although WIG1, also known as ZMAT3, is a target gene of p53, it could also activate Fas by directly combining to the 3’ UTR of Fas independent of p53, thereby promoting cell apoptosis^24^. Next, by using western blot and luciferase reporter assays, AGO2, LASP1, MAFG, and WIG1 were proven to be the target genes of miR-184, from which only WIG1 was validated as the functional target to improve the expression of Fas on HTR8 cells, consequently providing a novel target gene of miR-184.

MicroRNA agomir, a more stable miRNA agonist, can significantly increase the expression of miRNA by simulating endogenous miRNA^25^. Finally, we verified the role of miR-184 in vivo via overexpressing miR-184 in female BALB/c mice, and found miR-184 overexpression in vivo could promote the pregnancy loss. The underlying mechanism of pregnancy loss may be partly due to the apoptosis of trophoblast cells induced by miR-184. A recent article has found that PKP2 deficiency contributes to the hypermethylation of the CpG sites at miR-184 promoter, thus downregulating the expression of miR-184^26^. Hence, this provides an idea of epigenetic regulation for exploring the regulation mechanism of miR-184 during pregnancy period in the future.

Taken together, our data suggest that miR-184 is highly expressed in RSA, which targets WIG1 to promote the apoptosis of trophoblast cells via up-regulating Fas expression, thereby affecting the maintenance of normal pregnancy.
